# Hedgehog Pathway Inhibitors: Clinical Implications and Resistance in the Treatment of Basal Cell Carcinoma

**DOI:** 10.7759/cureus.13859

**Published:** 2021-03-12

**Authors:** Suzanne Habashy, Aliya Jafri, Hiba O Osman, Neena E Thomas, Somtochi Udekwe, Stacey E Heindl

**Affiliations:** 1 Family Medicine/Dermatology, California Institute of Behavioral Neurosciences & Psychology, Fairfield, USA; 2 Biochemistry and Family Medicine, California Institute of Behavioral Neurosciences & Psychology, Fairfield, USA; 3 Biochemistry, Jinnah Sindh Medical University, Karachi, PAK; 4 Internal Medicine, California Institute of Behavioral Neurosciences & Psychology, Fairfield, USA; 5 Psychiatry and Behavioral Sciences, California Institute of Behavioral Neurosciences & Psychology, Fairfield, USA; 6 Internal Medicine, Endocrinology, Pediatrics, Gynecology, Family Medicine, California Institute of Behavioral Neurosciences & Psychology, Fairfield, USA; 7 Medicine, California Institute of Behavioral Neurosciences & Psychology, Fairfield, USA; 8 Medicine, Avalon University School of Medicine, Willemstad, CUW

**Keywords:** hedgehog pathway inhibitor resistance, basal cell carcinoma, hegehog pathway inhibitors, vismodegib, sonidegib, hedgehog pathway

## Abstract

Basal cell carcinoma (BCC) is the most common non-melanoma skin cancer and is on the rise. Most BCCs are benign; however, a very small percentage are locally advanced and metastatic. The pathway that normally regulates cell growth and proliferation is directed by the hedgehog pathway (HP). In BCC, it becomes over-stimulated due to genetic abnormalities. Treatments for BCC include local treatment by cryotherapy (liquid nitrogen), topical immunosuppression, surgery, or radiotherapy. Systemic treatment may be required in locally advanced lesions, metastatic BCC, or individuals who are inoperable.

The systemic treatments of BCC act to inhibit the HP and are called hedgehog pathway inhibitors. The first one being vismodegib and the second sonidegib.

Although these treatments have shown promising results, they have prominent side effects in almost all patients, with few patients having to discontinue the treatment. About 50% of patients did not respond to treatment from the beginning, some had partial responses, others had recurrence after discontinuing the drugs, and few had worsening of the disease. In this paper, we will explore the most common side effects, resistance, and different methods to overcome resistance to ensure the highest rate of cure for BCC.

## Introduction and background

Skin cancer is the most common malignancy worldwide, with 3.5 million people diagnosed with non-melanoma skin cancer (NMSC) each year. Of this vast number, 80% of the cases are basal cell carcinomas (BCCs) [1]. Most BCCs are slow growing with a benign course. Around 0.8% are locally advanced (LBCC) and 0.4% are metastatic (MBCC) [1]. Considering that 80% of BCCs appear on the head and neck, locally advanced lesions can cause disfigurement and loss of function after excision [2]. Incidences of BCC are increasing by 10% per year, and the overall prevalence is higher in people with fair skin, light eyes, light hair color, and inability to tan. The risk is directly increased from the cumulative effect of ultraviolet light as well [1]. Severe ultraviolet radiation B (UVB) exposure causes DNA damage which promotes mutations in the tumor suppressor gene patched (PTCH). This further suppresses the hedgehog pathway (HP), allowing for cell growth dysregulation and immunosuppression, leading to the development of BCC. This is the theory of the development of BCC in Gorling syndrome or nevoid BCC and is the pathway on which few drugs work to treat BCC. Because of disruptions in this pathway, patients also have higher risks of developing other malignancies, such as medulloblastoma, rhabdomyosarcoma, benign ovarian cysts, cardiac fibromas, and mesenteric cysts. New studies also suggest other genetic mutations involved in the development of BCC [1].

The mainstay of BCC treatments includes surgical local excision, microscopic Mohs surgery, local destructive measures by cryotherapy, topical imiquimod, 5-fluorouracil, and photodynamic therapy, with most treatments having a five-year cure rate of ≥95% [2-4]. For LBCC and MBCC, surgical or local treatments are not curable and systemic therapies should be considered [3].

Vismodegib, the first oral systemic hedgehog pathway inhibitor (HPI) approved by the Food and Drug Administration (FDA) and European Medicines Agency (EMA), has become the first-line drug of choice for which radiotherapy and surgery are not recommended in LBCC and MBCC [2]. Sonidegib is the second HPI that has also been approved by the FDA. The efficacy and long-term safety of HPIs have been studied and explored, resulting in guidelines for the treatment and follow-up of patients regarding side effects, toxicity, tolerability, and quality of life [5]. Due to these inquiries about the drugs in question, resistance and poor or partial response to HPIs have been noted, mostly from gene mutations of the PTCH, the smoothened head (SMO), or bypassing the HPs entirely [6]. In this paper, we explore the use of HPIs in terms of their pathway mechanisms, side effects, tolerability, resistance, and methods to maximize their effects.

## Review

Understanding the hedgehog pathway

To understand the mechanism of vismodegib in BCC, we must first learn and understand the regular HP. The HP regulates normal cell development and proliferation. Disturbance of the pathway and disruptions of the gene itself are both causes of cancer development. The HP involves the hedgehog genes Sonic, Indian and Desert, the two PTCH genes, PTCH1 and PTCH2, and three GLI genes, GLI1, GLI2, GLI3 [6].

The hedgehog stimulating pathway is important in regulating proliferative and regulatory cellular development. Impairment of the genes mentioned and their pathways can beget tumors [6].

One gene of significance is the PTCH gene identified on 9p22.3. The PTCH protein product of the human PTCH gene is the sonic hedgehog (SHH) receptor, a protein in the integral membrane with 12 predicted transmembrane regions. This PTCH protein then binds a membrane g-protein receptor named SMO, thereby suppressing its action. SMO suppression is crucial for normal cell regulation and proliferation [6].

However, in BCC, due to gene mutations, the activated SMO inhibits the negative regulator of the suppressor of the fused protein (SUFU) which binds the GLI glioma-associated transcription factor in the cytoplasm [1]. Once SMO activates factor GLI, GLI increases transcription of PTCH, TGFB, BCL2, and GLI itself. The loss of this negative autoregulation and the downstream cellular changes stimulate cell proliferation and differentiation [6]. Activation of the SHH signaling (by the inactivating mutation of PTCH or activating mutations of SMO) is the key to developing BCC [6].

Such stimulation and activation are also found in other tumors such as medulloblastoma, ovarian and cardiac fibroma, Gorling syndrome, bladder carcinoma, and squamous cell carcinoma of the esophagus. Gorling syndrome is an early onset of multiple BCC, in addition to skeletal abnormalities, jaw cysts, macrocephaly, and palmoplantar pits [6].

Uncontrolled activation of the HP is evident in 95% of sporadic BCC in addition to other malignancies due to mutated PTCHAD1, while 10% are due to mutations of SMO [5].

The HPIs are effective in treating MBCC and LBCC in the following cases: disfiguring surgery, loss of vital function, and metastasis [1].

Hedgehog pathways inhibitors

Vismodegib is a first-generation synthetic molecule that binds to the SMO receptor, thereby inhibiting its action and inhibiting tumor growth [7]. It was first approved in 2012 by the FDA and in 2013 by the EMA, shortly followed by another drug in the same class, sonidegid, in 2015. Although both drugs are chemically different, they both act on the same pathway [7].

Vismodegib has been approved for the treatment of MBCC and LBCC, while sonidegib has been approved for the treatment of LBCC only [7].

In terms of dosing, vismodegib is given at a dose of 150 mg once daily and sonidegib is given at a dose of 200-800 mg/day until disease progression, unacceptable toxicity, withdrawal of consent, or death due to other reasons has occurred [8].

Response to hedgehog pathways inhibitors

Two major trials have been conducted for the overall response, effectiveness, and side effects of the HPI. In the primary analysis of the ERIVANCE study, all patients experienced treatment emergent side effects (TESE), while the TESE recorded in the STEVIE trial were 98% [8]. The results of the TESE of these studies are depicted in Table 1.

**Table 1:** Side effects of the two major trials for the use of vismodegib in BCC treatment. SCC: squamous cell carcinoma; BCC, basal cell carcinoma

Name of the trial	ERIVANCE study	STEVIE trial
Type of trial	Phase two, single-arm multicenter trial	Open-label trial including 36 centers and 167 sites
Muscle spasm	71.2%	62%
Alopecia	66.3%	62%
Dysgeusia	55.8%	54%
Weight reduction	51.1%	33%
Fatigue	43%	27%
Nausea	32.2%	13%
Decrease in appetite	28%	25%
Diarrhea	27%	17%
SCC	Less than 5%	4.2%

Other side effects include irregular menses, amenorrhea in women of childbearing potential, pneumonia, general health deterioration, and dehydration [8].

Most TESE noted in both studies were graded at levels one and two in severity, grade three occurred in 11%, and grade four occurred in 2%. The cumulative and chronic nature of the side effects resulted in discontinuation of the treatment by patients (approximately 31%) [8].

The incidence of TESE was higher during the first year of treatment and continued with prolonged use over 12 months, but no new TESE were observed or recorded after the first year of use [9].

Management of these effects are important to continue treatment, which includes calcium channel blockers or cyclobenzaprine in patients suffering from muscle spasms. Treatment interruption, for short periods, is a common practice to allow recovery from side effects and to allow patients to continue treatment, decreasing the chronicity of TESE [9].

Objective rate of response to hedgehog pathways inhibitors by the independent review facility

In the ERIVANCE study with vismodegib, the objective response rate (ORR) by independent review facility (IRF) was 30% in MBCC and 43% in LBCC [2]. ORR by investigator review was 45% in MBCC and 60% in LBCC [9]. At one year, updated ORRs increased from 30.3% to 33.3% in MBCC and from 42.9% to 47.6% in LBCC. At 30 months, the ORR was 48.5% in MBCC and 60.3% in LBCC [9].

These results are consistent with the STEVIE trial in which the ORR was 36.9% in MBCC and 68.5% in LBCC. In patients with Gorling syndrome, the ORR was 80% in MBCC and 81.7% in LBCC [8]. Long-term use of vismodegib at a low dose of 150 mg once a week has shown to reduce the risk of recurrence after complete regression of BCC compared to the 26.6% regression in patients who did not take the drug [10].

Vismodegib has also been used as a neoadjuvant therapy followed by surgery in high-risk BCC. In a new trial, the Vismoneo study showed that in patients with BCC of the scalp and face as well as inoperable or operable BCC with major aesthetic risks, the use of vismodegib reduced the surgical defect area by 27% and continued treatment for longer periods resulted in no recurrence. The same side effects were noted (muscle spasms, alopecia, dysgeusia); however, none were severe and all ceased after treatment withdrawal [11].

The health-related quality of life (HRQoL), using the Dermatology Life Quality Index (DLQI) in the six-month follow-up period after discontinuing vismodegib, has also been assessed. A high HRQoL value indicates better health outcomes, and due to its inverse relationship with the DLQI, the DLQI would then decrease. The results of the assessment showed that the recurrence of BCC at the six-month follow-up period had negative effects on patients, as the HRQoL was low and the DLQI increased [12].

Before beginning HPI treatment, it is imperative to assess the tumor in both the external/visible aspect and imaging for the infiltrating component with follow-up every three months. It is also important to stage the tumor according to the eighth TNM classification used by the Joint Committee on Cancer and Union of the International Cancer Control (UICC) [4]. For BCC of the head and neck, physical examination and imaging are used to assess the following: T (primary tumor), N (regional lymph node), and M (distant metastasis). For BCC of the perianal, vulva, or penis, physical examination should determine the T, while the N and M are determined by imaging only [4].

Imaging along with the response evaluation criteria in solid tumor (RECIST) and the modified RECIST (M REICST) are tools used to evaluate treatment response [13]. The RECIST takes colored photographs of the lesions with the plane of focus before treatment. This is done to identify the edge of the tumor, and in case the edges are not clinically visible, a plastic grid is used to provide additional visualization of the border. In subsequent clinic visits, the longest diameter is identified [7].

A complete response would show the disappearance of all lesions and pathological lymph node reduction in the short axis to ≤10 mm. A partial response is categorized as a ≥30% decrease in the sum of diameters of the target lesion. Stable disease is defined as no increase or decrease in the size of the tumor. A progressive response, which is progression of the lesion, is a ≥20% increase in the sum of the diameters and increase in the diameter by 5 mm or the appearance of a new lesion [4].

Resistance to hedgehog pathways inhibitors

In a study by Danial et al., primary resistance, in which the tumor never responded to treatment, occurred in 50% of the patients and secondary resistance, in which there was recurrence after the initial response to treatment, occurred in 20% of the patients [7]. Each category of resistance requires clinical examination and imaging studies for detection [7,4]. The cause of resistance was principally due to the mutation(s) of the SMO [7]. The primary resistance has been shown to exhibit mutations of the SMO G497W, while secondary resistance shows a pre and post-treatment mutation of the PTCH1, as well as mutations of SMO D473Y. In this mutation, the protein undergoes rearrangements resulting in partial obstruction of the protein drug entry [13].

The SUFU and negative regulators of the HP result in the activation of the GLI transcription factor and promote cell growth mutations [1]. The tumor cells reactivate the HP and resume growth after discontinuation of the HPI. This mutation is deemed as a class-related resistance as it occurs in both vismodegib and sonidegib [7]. The reason for this resistance is the tumor finding an alternative pathway that allows for its growth, despite blockage of the main and common pathway [13].

There are several mechanisms involved in bypassing the vismodegib and sonidegib inhibitory pathway and overcoming resistance. The first being arsenic trioxide as a potent HPI. It can bypass the SMO mutation by destabilizing the GL2. Itraconazole, an oral antifungal, is also an HPI as it decreases the mRNA in MBCC by reducing the mRNA expression of the GLI family. Using both of these drugs simultaneously can reduce HPI resistance by the following guidelines: intravenous arsenic oxide (0.3 mg/kg/d) for five days, every 28 days, for a total of three cycles, until unacceptable side effects occur; and with oral itraconazole 400 mg/d from day six to 28, for a total of three cycles, until unacceptable side effects or disease progression [14]. The effects of this combined treatment have been shown to suppress the HP by 75%, while vismodegib alone suppresses the HP by 90% [14].

Some patients showed tumor growth suppression after three months of treatment, but no tumor reduction. This may suggest that the treatment course could be prolonged to get a maximum and optimal response [14].

It is worth noting that the use of this combination therapy is effective in vismodegib naïve patients and it is not effective in patients previously treated with vismodegib as it has a poorer response [14].

Side effects of arsenic oxide and itraconazole are grade one and two leukopenia, increases in serum nitrogen and creatinine levels, transaminitis, dyspnea, and grade four leukopenia, an infection requiring antibiotic treatment, and asymptomatic atrial flutter that resolved spontaneously. Grade one side effects occurred in 95% of the patients, thereby causing 25% of the patients to cease treatment [14].

A different signaling pathway analysis identified was the phosphoinositide 3 kinase (PI3K/AKt) and cyclic nucleotide phosphodiesterases (PDEs). These stimulate tumor proliferation and allow for resistance to treatment. PDEs are enzymes that catalyze the degradation of a phosphodiester bond in cyclic adenosine monophosphate (cAMP) or cyclic guanosine monophosphate (cGMP) and are the potential molecular targets for treatment. Upon an increase in the cAMP, the SMO network becomes less branched. The nodes glycogen synthetase kinase 3B (GSK3B) and SUFU become less involved in conveying the signal from the SMO to the tumor proliferation endpoint, and the tumor proliferation endpoint is reduced. Therefore, cAMP is a potential network control point for reduction of the tumor proliferation, and strong inhibition of PDEs cause increases in cAMP [15].

**Figure 1:**
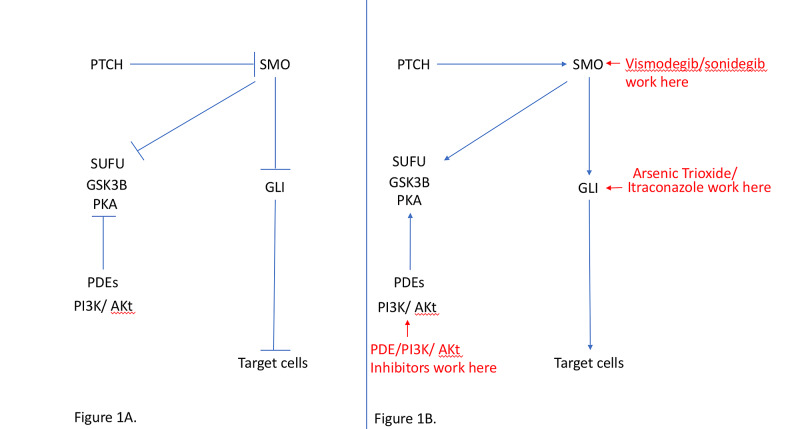
(A) Normal hedgehog pathway. (B) Site of action of different HPI on the hedgehog pathway. PTCH: patched; SMO: smoothened protein; SUFU: suppressor of the fused protein; GSK3B: glycogen synthetase kinase 3B; PKA: protein kinase A; GLI: transcription factor GLI; PDE: cyclic nucleotide phosphodiesterases; PI3K/Akt: phosphoinositide 3 kinase; HPI: hedgehog pathway inhibitors

A combination of PDE inhibition causing cAMP stimulation, with inhibition of SMO and the PI3K/AKT pathway, may be used to control the tumor proliferation. Figure 1 explains the HPI and the medications that overcome this resistance [15].

Anti-programed cell death protein 1 (PD-1) therapy was used for MBCC that progressed or were resistant to previous HPI treatment. PD-1 inhibitor compounds such as pembrolizumab and nivolumab are under investigation for the treatment of advanced BCC in clinical trials [14].

Gene expression has been identified in the HPs. A total of 16 genes have been recognized, the most prominent being GL11, Gl12, growth arrest specific 1 (GAS1), and WNT. GAS1 is a negative regulator of the HP. After treatment with vismodegib, the gene expression changes in all 16 genes, and the GAS1 persists at high levels after the treatment. Hence, GAS1 is considered a marker for the possible treatment response of LBCC [5]. However, it is lower in patients with complete recovery and higher in patients with partial recovery. Pre-treatment genetic testing is recommended to determine patients with possible resistance who might not benefit from HPI and can protect patients from side effect exposure and toxicity [16]. This genetic testing may also give us an idea about combination therapy to control tumor growth [15].

Other signaling pathways such as WNT, NOTCH, mTOR, and Hippo are regulated by non-coding micro RNA. Changes in the micro RNA expression are found with tumor progression and targeting micro RNA can reduce the resistance [1].

It is interesting to mention that recurrence after HPI discontinuation might not be due to resistance but may be explained by the persistence of a slow-cycling tumor cell population that is activating the WNT signaling pathway. The re-introduction of the HPI can achieve the same previous clinical response [4].

## Conclusions

The HPIs have been proven to be effective in treating LBCC and MBCC. The side effects observed in patients are mostly mild but may cause discontinuation from patients taking this treatment. Responses to these HPIs are variable, showing complete resistance, partial responses, recurrence, and even progression of these cancers. Resistance from certain HPIs may be overcome by acting on different modulators of the HP to switch off the tumor proliferation. Although few methods have been discovered to overcome such resistance, ongoing research is crucial to further explore these pathways and apply them in clinical practice.
